# A Robust Strontium Coordination Polymer with Selective and Sensitive Fluorescence Sensing Ability for Fe^3+^ Ions

**DOI:** 10.3390/ma16020577

**Published:** 2023-01-06

**Authors:** Zi-Wei Li, Bin Tan, Zhao-Feng Wu, Xiao-Ying Huang

**Affiliations:** 1 State Key Laboratory of Structural Chemistry, Fujian Institute of Research on the Structure of Matter, The Chinese Academy of Sciences, Fuzhou 350002, China; 2 College of Chemistry and Materials Science, Fujian Normal University, Fuzhou 350007, China; 3 Fujian Science & Technology Innovation Laboratory for Optoelectronic Information of China, Fuzhou 350108, China

**Keywords:** strontium, coordination polymer, fluorescence, Fe^3+^ sensor

## Abstract

Exploration of sensitive and selective fluorescence sensors towards toxic metal species is of great importance to solve metal pollution issues. In this work, a three-dimensional (3D) strontium coordination polymer of Sr_2_(tcbpe) (H_4_tcbpe = 1,1,2,2-tetrakis(4-(4-carboxy-phenyl)phenyl)ethene) has been synthesized and developed as a fluorescent sensor to Fe^3+^ ions. Sr_2_(tcbpe) shows a mechanochromic fluorescence with emission shifting from blue of the pristine to green after being ground. Notably, based on a fluorescence quenching mechanism, Sr_2_(tcbpe) displays a sensitive and selective fluorescent sensing behavior to Fe^3+^ ions with a detection limit of 0.14 mM. Moreover, Sr_2_(tcbpe) exhibits high tolerance to water in a wide pH range (pH = 3–13), demonstrating that Sr_2_(tcbpe) is a potential fluorescent sensor of Fe^3+^ in water.

## 1. Introduction

In the past two decades, great progress has been made in fluorescent coordination polymers (FL-CPs), which have been applied in bioimaging, light emitting, chemical sensing, and so forth [1,2,3,4,5]. Many fluorescence (FL) mechanisms have been developed in FL-CPs, exemplified by linker- or metal-centered emission energy transfer between metals and ligands (LMCT or MLCT) [6,7,8]. Based on the desired features of CPs, therefore, their fluorescence can also be custom-made by careful choice of inorganic metal cation and organic linker. The most important representatives are rare earth (RE)-based CPs, whose fluorescence is derived from RE metal centers. RE-based CPs have received intense attention due to their bright and narrow characteristic emission bands and long emission lifetime [9,10,11,12]. However, the scarcity of RE sources to some extent restricts their future applications. Alkaline earth (AE) metals such as Ca^2+^ and Sr^2+^, like RE cations, usually exhibit abundant and flexible coordination modes, making them good candidates as alternative metals to construct FL-CPs [13,14,15]. Because of their d^0^ electron configuration, AE^2+^ ions are very suitable to build FL-CPs with ligand-centered luminescence by using emissive organic linkers with a unique chromophore. AE metals are also good candidates to build FL-CPs because of their economic and environmental advantages. However, thus far, FL-CPs constructed from AE metals are comparatively rare [13,14,15].

Metal ions play essential roles in biological metabolism. However, metal species usually exist at a trace concentration level in biological systems, while an excess or deficiency of metals would bring great harm to the biological environment and even threaten life. For instance, excess Fe^3+^ can cause Alzheimer’s disease, while a lack of Fe^3+^ can result in hemochromatosis [16,17]. Therefore, many efforts have been devoted to monitoring the Fe^3+^ ions. A variety of FL-CP sensors towards Fe^3+^ ions have been developed in the past decade, which are mostly constructed from RE and transition metal (TM) ions [18,19,20]. However, as far as we know, AE-based FL-CPs with sensitive and selective Fe^3+^ sensing performance are comparatively rare [13,15].

Bearing this in mind, herein, we report a Sr-based FL-CP of Sr_2_(tcbpe) (H_4_tcbpe = 1,1,2,2-tetrakis(4-(4-carboxy-phenyl)phenyl)ethene). Sr_2_(tcbpe) exhibits an interestingly mechanochromic FL inherited from the tetraphenyl ethylene emitting center in H_4_tcbpe with aggregation-induced emission (AIE) characteristics. Sr_2_(tcbpe) shows FL sensing performance towards Fe^3+^ with good selectivity and sensitivity, representing the first FL Sr-CP sensor to Fe^3+^ ions. It also possesses an excellent tolerance to solvents and water even in acid/base conditions, indicating that Sr_2_(tcbpe) is a promising FL sensor to probe Fe^3+^ ions in water. 

## 2. Materials and Methods

Reagents. SrCl_2_ (≥99%, Adamas-beta, Shanghai Titan Chemical Co., Ltd., Shanghai, China); DMF (*N*,*N*-dimethyl-Formamide, AR, Greagent, Shanghai Titan Chemical Co., Ltd.); formic acid (≥98%, Adamas-beta, Shanghai Titan Chemical Co., Ltd.). H_4_tcbpe has been synthesized using a previously reported method [21].

Preparation of Sr_2_(tcbpe). A mixture of strontium chloride (50 mg) and H_4_tcbpe (20 mg) in 5 mL DMF, 2 mL H_2_O, and 500 μL formic acid was sealed into a 20 mL glass vial and heated at 120 °C for 2 days. Light-yellow block-like crystals were obtained by filtration after the vial was cooled to room temperature (50 mg, 30% yield based on strontium, Figures S1 and S2). Elemental analyses calculated for Sr_2_(tcbpe): C 61.76%, H 3.26%. Found: C61.20%, H 3.83%.

Physical measurements. Powder X-ray diffraction (PXRD) patterns were recorded on a Rigaku MiniFlex II diffractometer using CuK*α* radiation (*λ* = 1.54178 Å). A graphite monochromator was used and the generator power settings were set at 44 kV and 40 mA (Figure S3). Data were collected in a 2*θ* range of 3 and 35° with a scanning speed of 1.0°/min. Thermogravimetric (TG) data were collected on a NETZSCH STA449C thermogravimetric analyzer with a temperature ramping rate of 10 °C/min from 30 to 700 °C under nitrogen gas flow (Figure S4). Elemental analyses for C, H, and O were performed on a German Elementary Vario EL III instrument. Single crystal X-ray diffraction data were collected with graphite-monochromated MoK*α* (*λ* = 0.71073 Å) using an XtaLAB Synergy R, HyPix diffractometer at 298(2) K.

FL measurements. The as-made crystalline samples of Sr_2_(tcbpe) were manually ground to obtain a fine powder. A 2 mg powdered sample was dispersed in 2 mL of the given organic solvents or 10^−2^ M metal ion solution by ultrasonication to obtain stable FL suspensions. The FL emulsion was then placed in a 1 cm width quartz cell to record the FL spectra using a PerkinElmer LS55 FL spectrometer. The FL detection experiments were carried out by adding varied amounts of 0.5 × 10^−2^ M Fe^3+^ ions into the prepared suspensions with a pipette. For all FL measurements, the excitation wavelength was monitored at 380 nm and the corresponding emission wavelengths were monitored from 400 nm to 700 nm.

Stability measurements. A 15 mg as-made crystalline sample was immersed in 2 mL of organic solvents or water with a range of pH values over 24 h. Then the immersed samples were collected by filtration and used for further PXRD measurements.

X-ray crystallography. A single crystal suitable for single-crystal X-ray diffraction (SCXRD) was selected under an optical microscope and glued to a thin glass fiber. The structure was solved by direct methods and refined with full-matrix least-squares techniques using the *SHELX*2018 package [22]. The CCDC number for Sr_2_(tcbpe) is 2224915. The detailed crystallographic data and structure-refinement parameters are summarized in Table 1.

## 3. Results and Discussion

### 3.1. Crystal Structure Analysis

**Figure 1:**
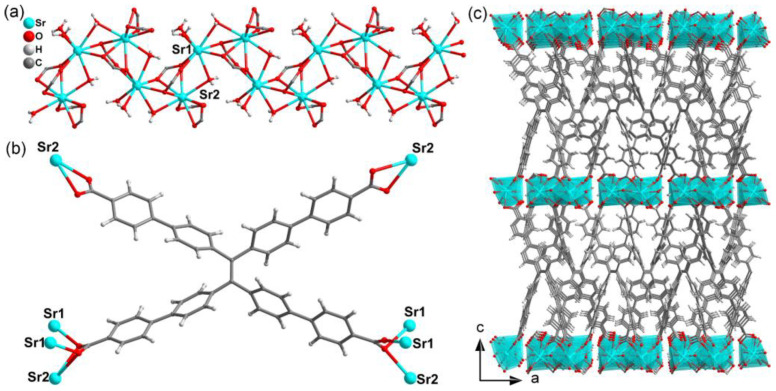
(**a**) The coordination environments of Sr^2+^ ions in the 1D chain-like SBU of structure Sr_2_(tcbpe). (**b**) The coordination mode of tcbpe^4−^ in Sr_2_(tcbpe). (**c**) The 3D skeleton of Sr_2_(tcbpe) seen from the *b*-axis.

Single-crystal analysis indicates that Sr_2_(tcbpe) displays a three-dimensional (3D) structure. As seen in Figure 1a, there are two crystallographically independent Sr^2+^ cations. Sr1 is eight-coordinated by four water molecules (of which two as bridging linkers and two as terminal molecules) and four monodentate coordinating carboxylic groups from four different tabpe^4−^ ligands (Figure S5a). Sr2 is nine-coordinated exhibiting similar coordination to RE ions. The Sr2 is connected with three carboxylic groups from three different tcbpe^4−^ ligands in chelating coordination, one carboxylic group in a monodentate coordinating mode, and two bridging water molecules (Figure S5b). The stretching vibration peak at ~3550 cm^−1^ for the –OH of the free carboxylic group in H_4_tcbpe has disappeared, which further demonstrated the coordination between the Sr^2+^ and tcbpe^4−^ ligands (Figure S2). The Sr1 and Sr2 are interconnected by one bridging water and one carboxylic group to form a 1D zigzag chain along the *b* direction as the secondary building unit (SBU, Figure S5c,d). Such 1D chain-like SBUs are bridged by tcbpe^4−^ ligand with only one coordination mode to connect the six neighboring 1D chains to generate a 3D nonporous structure (Figure 1b,c).

### 3.2. FL Studies

**Figure 2:**
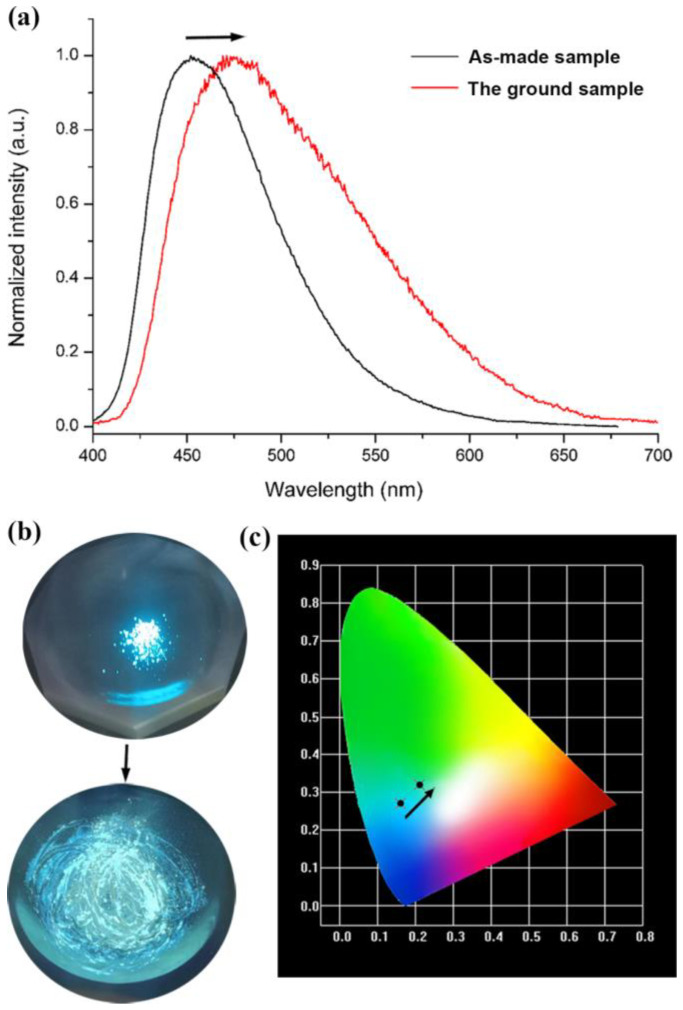
(**a**) The FL spectra of the as-made and ground samples of Sr_2_(tcbpe) measured at room temperature. (**b**) Photographic image of the luminescent changes of Sr_2_(tcbpe) under 365 nm light before and after grinding. (**c**) Photograph of the CIE chromaticity diagram for Sr_2_(tcbpe) before and after grinding.

**Figure 3:**
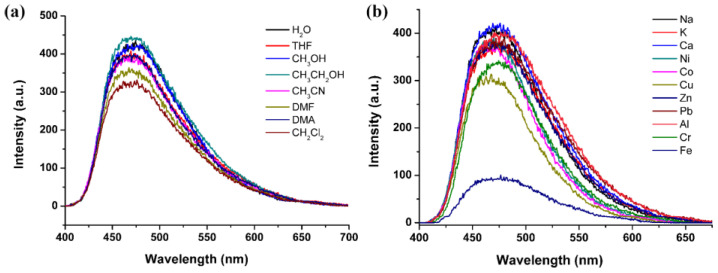
The FL of the powdered Sr_2_(tcbpe) dispersed in different solvents (**a**) and 0.5󠆼 × 10^−2^ M aqueous solution containing different metal ions (**b**).

H_4_tcbpe is a bright emissive ligand with aggregation-induced emission (AIE) [23,24]. Therefore, ligands bearing this AIE center of tetraphenyl ethylene usually show sensitive FL to external stimuli [23,24,25]. Herein, the AIE character is inherited by Sr_2_(tcbpe), which exhibits interestingly mechanochromic FL. As depicted in Figure 2a, the solid-state FL spectrum of the as-made Sr_2_(tcbpe) shows a blue emission maximized at 470 nm when excited by 410 nm light (Figures S6 and S7). A mechanoresponsive bathochromic shift in FL emission after grinding is observed—that is, a shift from the blue emission centered at 470 nm to a green emission maximized at 485 nm (inset in Figure 2a, Figures S7 and S8, ESI). Consequently, the chromaticity coordinate for the as-made sample is (0.16, 0.27), while that for the ground sample is (0.21, 0.32) (Figure 2b). When dispersed in various lab solvents (e.g., *N*,*N*-dimethylformamide (DMF), *N*,*N*-dimethylacetamide (DMA), acetone, and ethanol), the powdered Sr_2_(tcbpe) exhibits no solvent-dependent FL (Figure S9), which showed, to some extent, a quenching of FL intensity (Figure 3a). The powdered samples of Sr_2_(tcbpe) were dispersed in various 0.5󠆼 × 10^−2^ M *M*(AC)*_n_* solutions (*n* = 1–3, *M* = Na^+^, K^+^, Ca^2+^, Zn^2+^, Cu^2+^, Co^2+^, Ni^2+^, Pb^2+^, Fe^3+^, Al^3+^, and Cr^3+^) to test their FL-sensing selectivity to metal ions. As can be seen in Figure 2b, although different metal ions exhibit mild FL-quenching effects to Sr_2_(tcbpe), only the Fe^3+^ ion almost entirely quenches its FL, demonstrating that the material is a selective FL sensor to Fe^3+^ ions.

**Figure 4:**
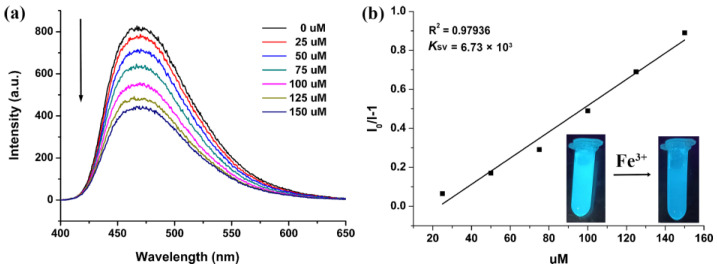
(**a**) FL of Sr_2_(tcbpe) with the addition of 0.5󠆼 × 10^−2^ M Fe^3+^ ions in an increasing amount. (**b**) The corresponding *K*_sv_ curve. Insets show the FL photographs of Sr_2_(tcbpe) before and after the addition of Fe^3+^ ions.

The FL-quenching percentage was quantitatively monitored by the addition of different amounts of 0.5󠆼 × 10^−2^ M Fe^3+^ ions into the FL emulsion (2 mg Sr_2_(tcbpe) dispersed in 2 mL H_2_O). The FL intensity of Sr_2_(tcbpe) is gradually quenched with increasing Fe^3+^ ion content, and the FL was quenched by almost 50% at a concentration of 0.1 mM Fe^3+^ ions (Figure 4a). As shown in Figure 4b, the Stern–Volmer equation (*I*_0_/*I* = 1 + *K*_sv_[M], in which *I*_0_ and *I* are the FL intensity of Sr_2_(tcbpe) without and with the addition of Fe^3+^, and [M] is the molarity of Fe^3+^ and *K*_sv_ is the quenching constant), exhibits good linear behavior. The value of *K*_sv_ was found to be 6.73 × 10^3^ M^−1^. The limit of detection was obtained as 0.14 mM from the ratio of 3*δ*/slope. The detection sensitivity to Fe^3+^ ions is even comparable to that of porous FL-CP sensors [26,27,28,29,30,31,32,33,34,35,36,37,38,39], Table S1. 

**Figure 5:**
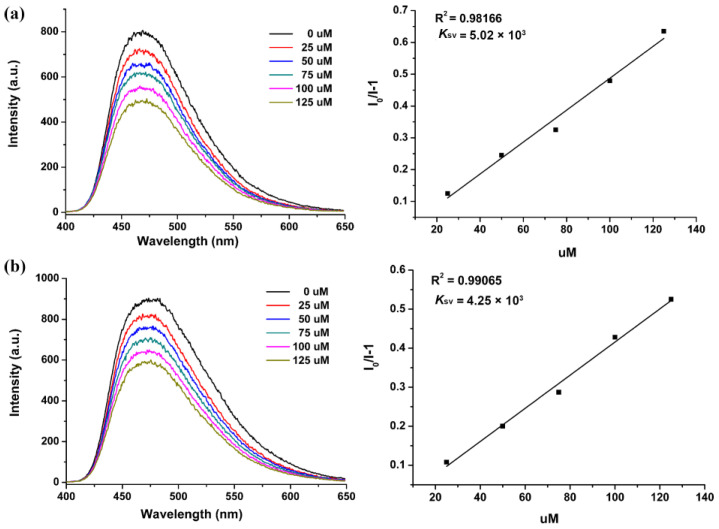
(**a**) FL spectra of Sr_2_(tcbpe) dispersed in 0.5󠆼 × 10^−2^ M Na^+^ aqueous solutions by adding Fe^3+^ ions at various concentrations and the corresponding *K*_sv_ curve. (**b**) FL spectra of Sr_2_(tcbpe) dispersed in 0.5 × 10^−2^ M Ca^2+^ solutions with the addition of Fe^3+^ ions and the corresponding *K*_sv_ curve.

Selectivity is an important parameter for FL sensors. Na^+^, Ca^2+^, etc. are usually coexisting ions in water in nature. Therefore, the powdered sample of Sr_2_(tcbpe) was dispersed in separate 0.5󠆼 × 10^−2^ M Na^+^ and Ca^2+^ aqueous solutions to check the sensing selectivity towards Fe^3+^. As depicted in Figure 5, the FL intensity of Sr_2_(tcbpe) in these interferential metal ions showed a similar quenching response to Fe^3+^ as that of the FL emulsion dispersed in water. The decrease in FL intensity also exhibits a good linear relationship with Fe^3+^ concentration. The *K*_sv_ values are 5.02 × 10^3^ and 4.25 × 10^3^ M^−1^ for Fe^3+^ ions (Insets of Figure 5), respectively, which are comparable to that in water. The results demonstrate that Sr_2_(tcbpe) possesses a good sensing selectivity toward Fe^3+^ even in water systems with various interferential metal ions.

The as-made Sr_2_(tcbpe) also exhibits excellent solvent- and water-tolerances even in acid/base conditions. The prepared compound has exhibited good anti-solvent stability, as demonstrated by a comparative study of the PXRD patterns for the samples before and after immersion in common lab solvents (Figure S10). As shown in Figure S11, the PXRD patterns of the samples immersed in water with pH values of between 3 to 13 remain the same as the simulated pattern, indicating that the skeleton of Sr_2_(tcbpe) is still maintained in acid or base water environments. The good selectivity and chemical stability make Sr_2_(tcbpe) a promising FL sensor for Fe^3+^ in water. Fe^3+^ ions due to their d^5^ configuration possess a strong electron-withdrawing ability. The UV light-excited electrons of Sr_2_(tcbpe) transfer to the Fe^3+^ ions, thus resulting in a decrease in the FL intensities of the Sr_2_(tcbpe) [28,29,30,31]. 

## 4. Conclusions

An AIE-emitting ligand of H_4_tcbpe has been coordinated with Sr^2+^ to assemble an FL Sr-CP. The obtained compound, Sr_2_(tcbpe), exhibits a mechanoresponsive FL shifting from blue to green emission. Remarkably, the water-stable Sr_2_(tcbpe) represents the first FL Sr-CP sensor for Fe^3+^ ions with good sensitivity and selectivity. More AE-based FL-CPs sensors designed to detect toxic species will be created in our lab in the future. 

## Figures and Tables

**Table 1:** Crystallographic data and structural refinement details for Sr_2_(tcbpe).

Empirical formula	Sr_2_C_54_H_40_O_12_
Formula weight	1056.10
Crystal system	Monoclinic
Space group	*P*2_1_
*T*/K	298(2)
*λ*/Å	0.71073
*a*/Å	10.0613(2)
*b*/Å	9.8710(2)
*c*/Å	22.7537(3)
*β*/º	90.0016(11)
*V*/Å^3^	2259.79(7)
*Z*	2
*D_c_*/Mg·m^−3^	1.552
*μ*/mm^−1^	2.428
*F*(000)	1072
Measured refls.	50,879
Independent refls.	11,257
*R*_int_	0.0504
No. of parameters	638
*GOF*	1.038
Flack parameter	0.401(10)
^a^*R*_1_, ^b^*wR*_2_ [*I* > 2*σ*(*I*)]	0.0490, 0.1226
^a^*R*_1_, ^b^*wR*_2_ (all data)	0.0516, 0.1238

^a^*R*_1_ = ∑║*F*_o_│ − │*F*_c_║/∑│*F*_o_│. ^b^ *wR*_2_ = {∑*w*[(*F*_o_)^2^ − (*F*_c_^2^)]^2^/∑*w*(*F*_o_^2^)^2^}^1/2^.

## Data Availability

Not applicable.

## Supplementary Materials

The following supporting information can be downloaded at: https://www.mdpi.com/article/10.3390/ma16020577/s1. Figure S1: Photograph of the as-made sample of Sr_2_(tcbpe); Figure S2: IR spectra for the H_4_tcbpe ligand and the as-made sample for Sr_2_(tcbpe); Figure S3: Experimental PXRD pattern of Sr_2_(tcbpe) compared with the simulated pattern; Figure S4: TG curve of the as-made Sr_2_(tcbpe); Figure S5: Extra structural details for Sr_2_(tcbpe); Figure S6: UV-Vis absorption spectrum for the as-made Sr_2_(tcbpe); Figures S7 and S8: FL spectra for Sr_2_(tcbpe) before and after grinding; Figure S9: Photographs of the powdered Sr_2_(tcbpe) dispersed in solvents excited at 365 nm UV light; Figures S10 and S11: PXRD patterns of the sample immersed in different lab solvents and water in a wide range of pH (3–13); Table S1: Comparison of *K*_sv_ for the reported CPs FL sensors for Fe^3+^ ion.
