# Supplementary material for: A Robust Strontium Coordination Polymer with Selective and Sensitive Fluorescence Sensing Ability for Fe3+ Ions
Source: Materials (Basel). 2023 Jan 6;16(2):577. doi: 10.3390/ma16020577 (PMC9866177; doi:10.3390/ma16020577)
Supplement: Supplementary file 1 [file materials-16-00577-s001.zip › materials-2129668-supplementary.pdf]

materials

A robust strontium coordination polymer with selective and sensitive fluorescence sensing ability for Fe^{3+} ions

Zi-Wei Li [1,2], Bin Tan [1,3], Zhao-Feng Wu [1,3,*] and Xiao-Ying Huang [1,*]

[1] State Key Laboratory of Structural Chemistry, Fujian Institute of Research on the Structure of Matter, The Chinese Academy of Sciences, Fuzhou 350002, China
[2] Fujian Normal University, 32 Shangsan Road, Fuzhou 350007, China
[3] Fujian Science & Technology Innovation Laboratory for Optoelectronic Information of China, Fuzhou 350108, China
[*] Correspondence: zfwu@fjirsm.ac.cn (Z.-F.W.); xyhuang@fjirsm.ac.cn (X.-Y.H.); Tel.: +591-63173146 (X.-Y.H.)

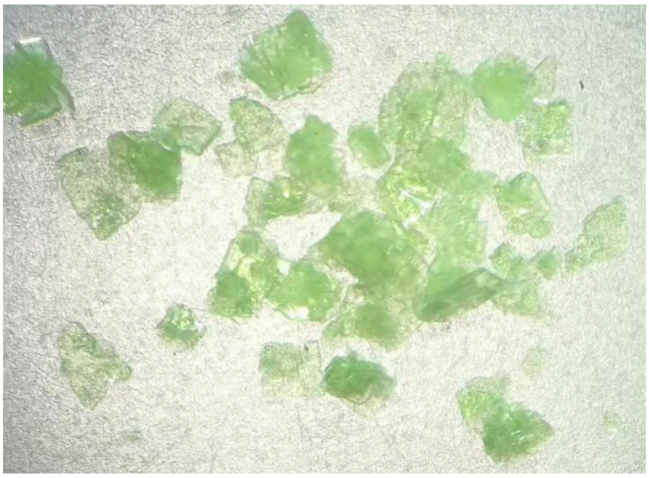

Figure S1. The photograph of the as-made sample for Sr_2(tcbpe).

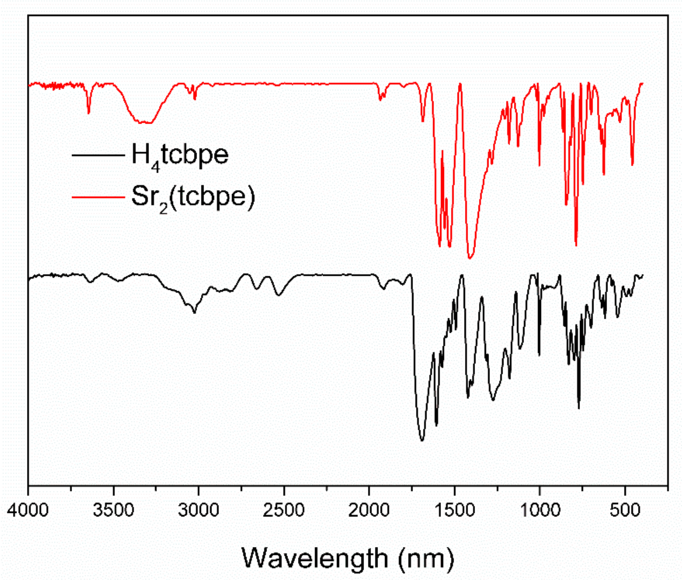

Figure S2. The IR spectra for the H_4tcbpe ligand and the as-made sample for Sr_2(tcbpe).

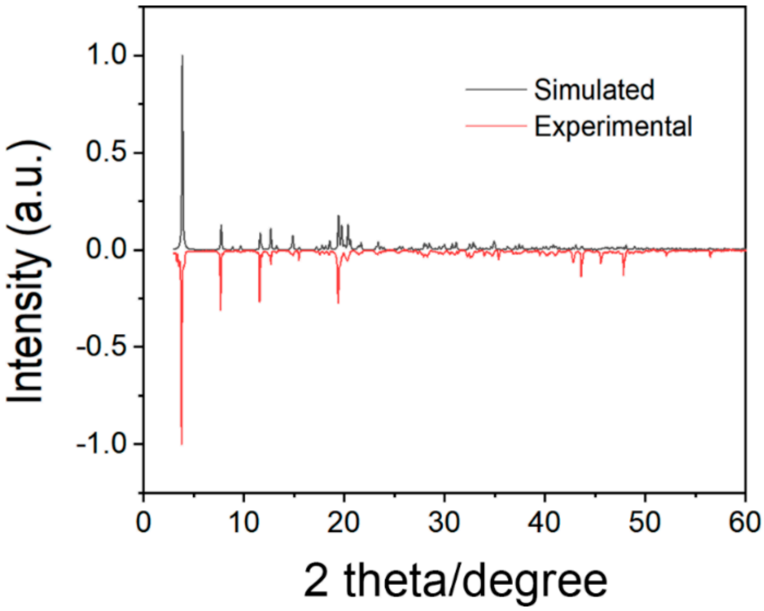

Figure S3. Experimental PXRD pattern of Sr_2(tcbpe) compared with the simulated one.

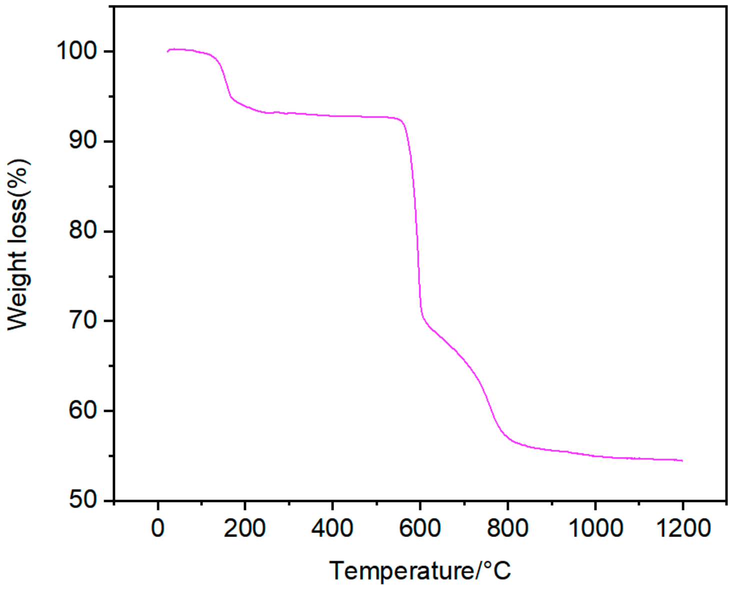

Figure S4. TG curve of the as-made sample of Sr_2(tcbpe).

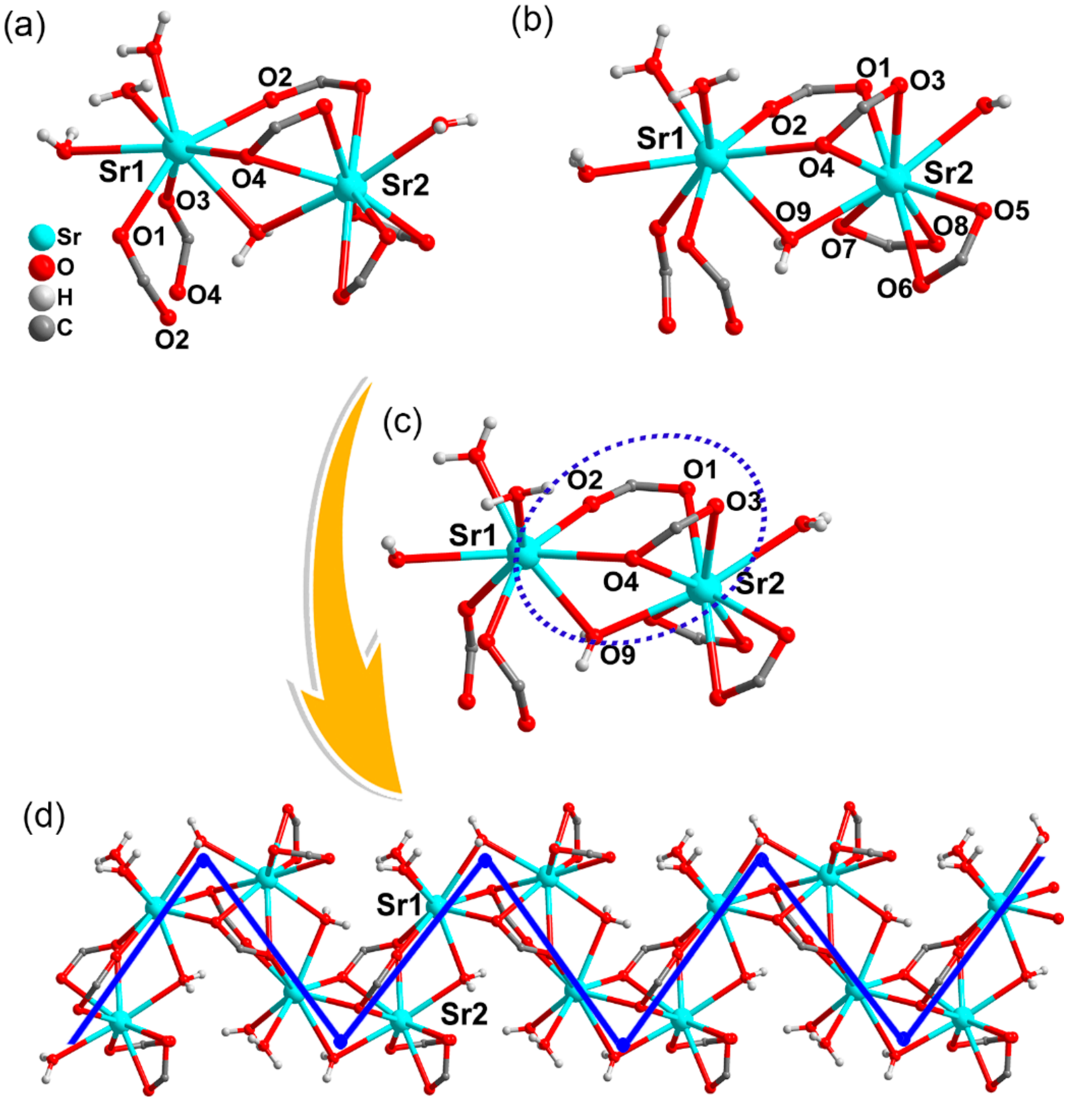

Figure S5. The coordination environments of Sr1 (a) and Sr2 (b) atoms. (c) The coordination environments of carboxylic groups and water that bridge Sr1 and Sr2 which has been highlighted in dotted blue circle. (d) The zigzag like 1D chain in Sr_2(tcbpe).

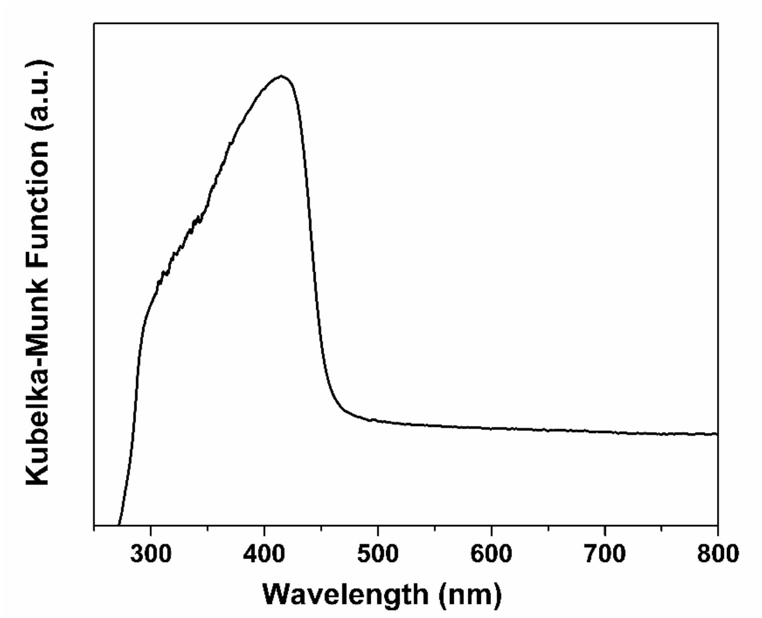

Figure S6. The UV-Vis absorption spectrum for the as-made Sr_2(tcbpe).

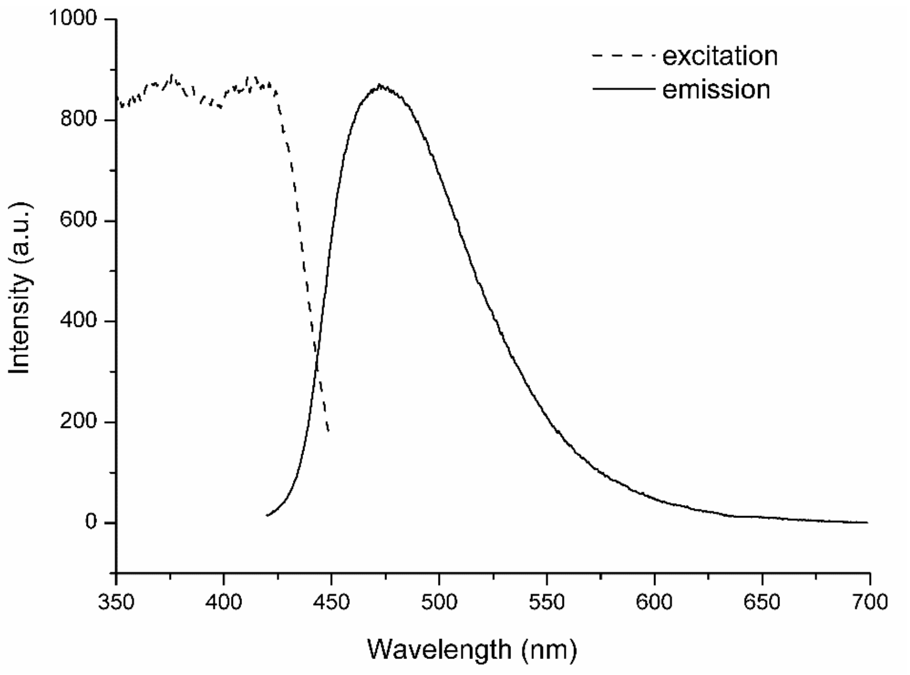

Figure S7. The FL spectra for the as-made Sr_2(tcbpe). The excitation and emission bands are depicted as dotted and solid line, respectively.

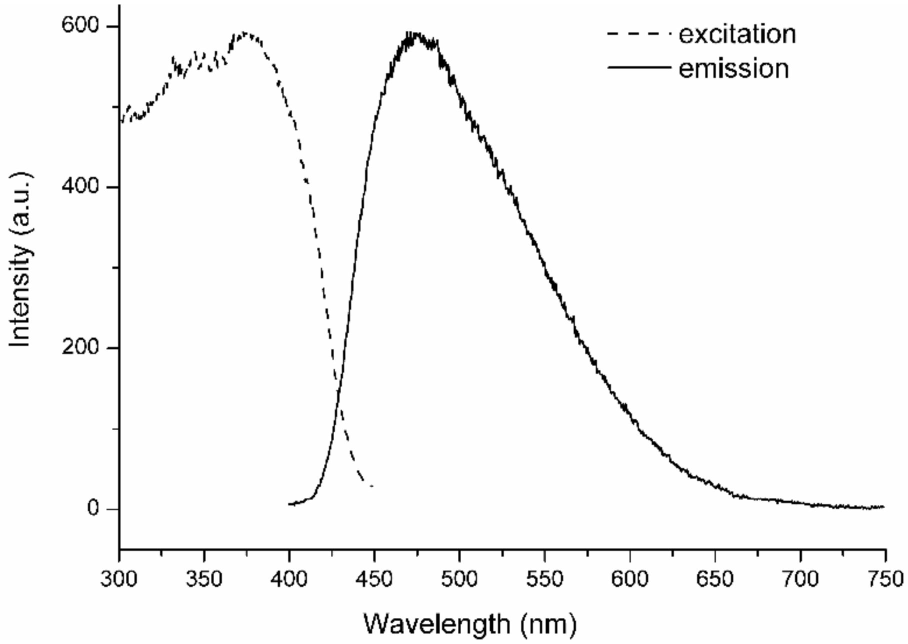

Figure S8. The FL spectra for the Sr_2(tcbpe) after being ground. The excitation and emission bands are depicted as dotted and solid line, respectively.

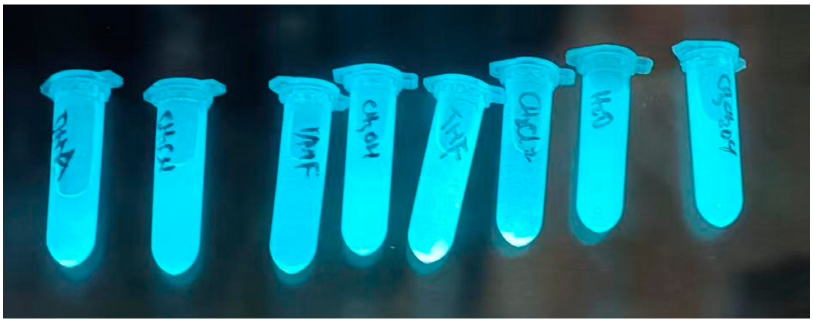

Figure S9. The photographs of the powdered Sr_2(tcbpe) dispersed in various solvents at 365 nm UV light.

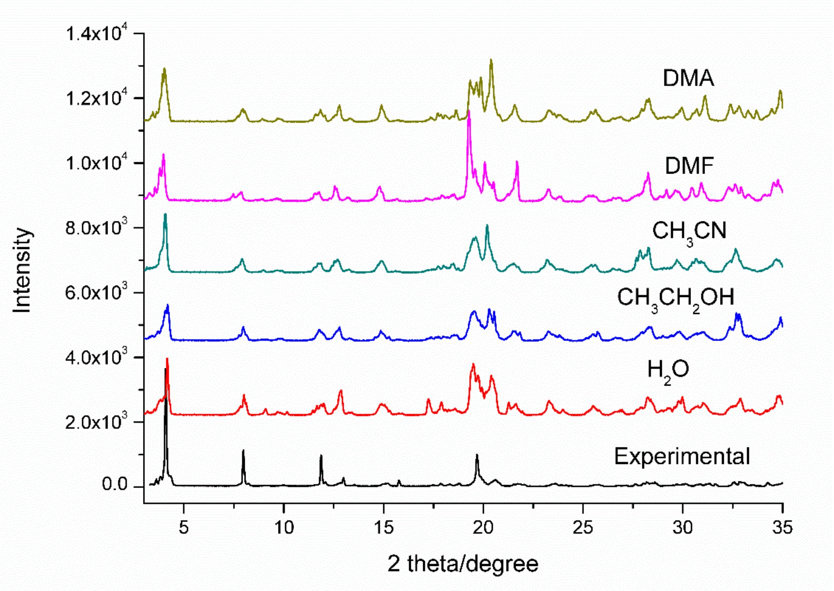

Figure S10. PXRD patterns of the sample immersed in different lab used solvents for 24 hours.

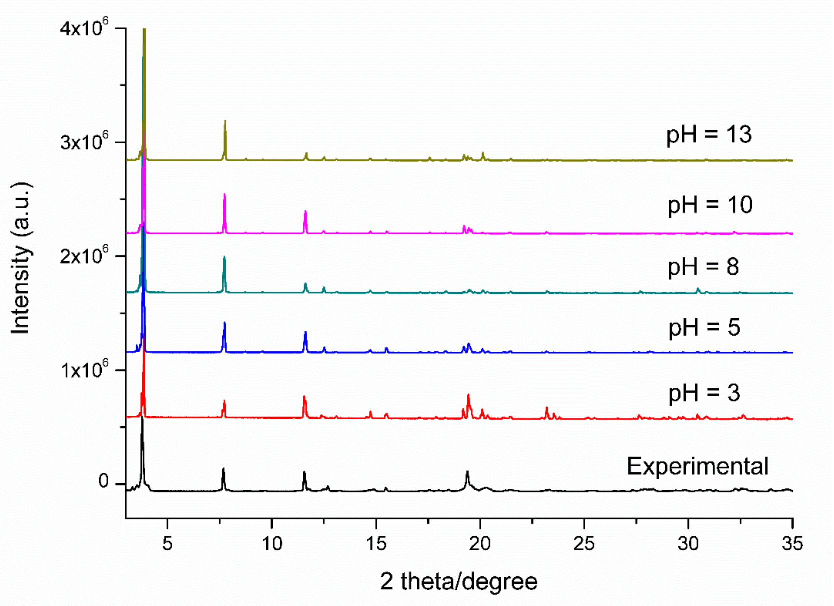

Figure S11. PXRD patterns of the sample immersed in water with different pH values for 24 hours.

Table S1. Comparison of K_{sv} for the reported CPs FL sensors for Fe^{3+} ion.

Compound	Dispersed solvents	K_{sv} (M^{-1})	references
Sr_2(tcbpe)	H_2O	6.73×10^3	This work
FJI-C8	DMF	8.25×10^3	26
[Me_2NH_2][In(abtc)]·solvents	DMF	3.49×10^3	27
[$Mg_2Zn_2(OH)_2$(1,4-NDC)$_3$(H_2O)$_2$]·6H_2O	ethanol	1.70×10^4	29
[Cd(p-CNPhHIDC)(4,4′-bipy)$_{0.5}$]	H_2O	1.99×10^3	31
[Zn(p-CNPhHIDC)(4,4′-bipy)]		1.37×10^3	
$Zn_2(NO_3)_2$(4,4′- bpy)$_2$(TBA)	H_2O	7.48×10^3	32
$Zr_6O_4(OH)_4$(2,7-CDC)$_6$]·19H_2O·2DMF	H_2O	5.5×10^3	33
Al-MIL-53-N_3	H_2O	6.13×10^3	34
[Zn_2(tpeb)(bpdc)$_2$]	H_2O	1.326×10^4	35
Zn(L)$_2$	H_2O	1.34×10^4	36
Zn(L)(DBT)	DMSO+H_2O	1.19×10^4	37
[Cd_2(L)$_2$(bpe)$_2$]·3DMF·2.5H_2O	DMF	1.74×10^4	38
[Cd(5-asba)(bimb)	H_2O	1.78×10^4	39
